# 

**Published:** 1829-03

**Authors:** 


### MONTHLY LIST OF MEDICAL BOOKS.

[*Medical Works cannot be entered on this List except a copy be sent for the purpose; the titles of Books having frequently been transmitted to us, as published, which have not appeared for weeks, or even months, after.*]

A New System of Treating the Human Teeth; explaining the Causes which lead to their Decay, and the most approved Methods of preserving them. To which is added, some Account of a Discovery made by the Author for the Cure of Toothach and Tic Douloureux, &c. By J. P. CLARK, A.M. Dentist. —8vo. pp. 163. Longman, London.

The Influence of Physical Education in producing and confirming in Females Deformity of the Spine. By E. W. DUFFIN, Surgeon.—8vo. pp. 135. London, 1829.

A Manual for the Use of Students preparing for Examination at Apothecaries' Hall. By JOHN STEGGALL, M.D.—12mo. pp. 260. Anderson, 1829.

The Anatomy and Physiology of the Nervous System. By VALENTINE FLOOD, A.M. M.B. &c. Vol. I.—Dublin, 1828.

A General, Medical, and Statistical History of the present Condition of Public Charity in France; comprising a detailed Account of all Establishments for the Sick, the Aged, and the Infirm; for Children and for Lunatics, &c. By DAVID JOHNSTON, M.D. &c.—8vo. pp. 605. Edinburgh; and Simpkin and Marshall, London.

Selections from Physicians' Prescriptions; containing, 1, a copious List of Forms, Phrases, and Abbreviations used in Prescriptions, with Notes; 2, a Series of Prescriptions, illustrating the use of these Forms; 3, the Grammatical Construction of Prescriptions. For the use of Medical Students. By J. PEREIRA, F.L.S. &c. Fourth Edition, enlarged.—Highley, London, 1829.

An Essay on Mineral, Vegetable, Animal, and Aerial Poisons; classified according to Orfila; including the general Symptoms, Treatment, Tests, Morbid Appearances, &c. with the Means of restoring Suspended Animation. To which is appended, a Description of the Stomach-Pump. By JOHN STEGGALL, M.D.—18mo. Churchill, London, 1829.

A Treatise on the Diseases of the Chest, and on Mediate Auscultation. By R. T. H. LAENNEC, M.D. &c. From the French, with Notes and a Sketch of the Author's Life. By JOHN FORBES, M.D. &c. Plates. Third Edition, with additional Notes.—8vo. pp. 736. Underwood, 1829.

The American Journal of the Medical Sciences, November 1828.—Philadelphia.

Traité de l'Acupuncture, ou Zin-King des Chinois et des Japonais. Par J. M. CHURCHILL, Surgeon. Traduit de l'Anglais, par M. R. CHARBONNIER, M.D.—Paris.

To the interesting essay of the author, the translator has merely added a few brief notes.

Medical Botany. By J. STEPHENSON, M.D. &c. and J. M. CHURCHILL, F.L.S. &c. No. 25, for January 1829, containing Plates of the Rosa Canina, Crocus Sativus, Myroxylon Peruiferum, and Polygala Senega.—No. 26, for February, with Plates of the Myristica Moschata, Solidago Virgaurea, and Matonia Cardamomum.

Although the reputation of this work is now well established, the endeavours of the Editors to render it attractive have not diminished. The plates are elegantly executed; the descriptions of them are scientifically correct, and practically useful.

### NOTICES.

*In answer to* Dr. R. *and* Mr. M., *the Editors beg to refer to the statement at the head of the* Monthly List of Medical Books.

*The letter of* H. *is too personal for insertion in our Journal. The same appeal has been made to* Mr. HICKS, *by a Correspondent in the* Medical Gazette, *in a more proper, because in a more temperate manner.* Mr. HICKS *is bound to reply: if he does not, his professional brethren will justly complain.*

